# Special Issue “Optimising Interval Training Prescription”

**DOI:** 10.3390/sports10060087

**Published:** 2022-06-02

**Authors:** François Billaut

**Affiliations:** Department of Kinesiology, Laval University, Quebec, QC G1V 0A6, Canada; francois.billaut@kin.ulaval.ca

High-intensity interval training, the so-called HIT, was popularized among athletes in the 1980′s and has been shown to be one of the most effective training modalities for improving athletic performance in various sports. Extensive research has focused on understanding the acute and chronic responses to different forms of HIT to optimize the development of fitness in endurance, power, and team- and racquet-sports. A variety of cardiovascular, peripheral, and neural adaptations contribute to increasing cardiorespiratory fitness, power, and endurance after HIT programs. Endurance athletes will typically use the entire spectrum of intensity, duration, and repetition number to manipulate the training stimulus and target a broad range of adaptations in their yearly training plan. On the other hand, athletes in power-oriented sports will target a narrower selection of session types that often revolve around short and very short HIT to boost their aerobic fitness within specific periods of the annual plan. Although the responses to training have become better understood, sport scientists and coaches continue to explore innovative ways to increase the load (i.e., stress) imposed by these training sessions to further optimise and/or accelerate chronic physiological responses to exercise, and to produce greater athletic performance gains. The aim of this Special Issue is to provide practitioners with new evidence-based data about the use of external stimuli and/or the manipulation of training variables to enhance the “quality” of acute, chronic interval, and repeated-sprint training sessions for long-term adaptations in varied athletic populations.

The current Special Issue titled “Optimising Interval Training Prescription” published five original manuscripts discussing the relevance and pitfalls of the currently available training models, the ergogenic impact of nutritional strategies, and occlusion training modalities, as well as the prevalence of specific genes in athletes. A group from Montreal, Canada, revisited the popular Skiba and Coggan models that are widely used to prescribe HIT sessions in cycling [1]. Simulating more than 6000 HIT sessions with three different fictitious athletic profiles (time-trialist, all-rounder, and sprinter), the authors highlighted some inherent pitfalls to these models from logical deductions. They revealed several sessions that would require athletes to surpass their maximal power output over the exercise duration to be able to complete the prescribed session. These HIT sessions, deemed by the authors to be “impossible” to perform in real settings, were especially characterized by short exercise intervals performed in the severe and extreme intensity domains and separated by long recovery periods. The Skiba model yielded between ~4 and 23% of impossible sessions depending on the athlete profile, and the Coggan–Modified model yielded ~1 to 3%. Therefore, the study by Briand and colleagues [1] clearly highlighted the importance of understanding these models’ inherent limitations, and that the practitioner should exert caution and judgment when prescribing HIT based on these models.

Exercise capacity is determined by several factors, for example, maximal cardiorespiratory fitness, exercise economy, and anaerobic capacity. Importantly, these performance phenotypes are at least partly influenced by genetics, and one could argue that the prevalence of some genes could be related to physical fitness. In fact, research has shown that some key genes involved in skeletal muscle metabolism regulation are more prevalent in trained athletes than in the normal untrained population, and that having an overrepresentation of specific polymorphisms could lead to superior training responses. In the study by Ramírez de la Piscina-Viúdez et al. [2], the authors tested the hypothesis that the genotype distribution of the monocarboxylate transporter 1 (MCT1) A1470T polymorphism would differ between athletes and the general population, and that the MCT1 A1470T polymorphism might influence high-intensity exercise performance in endurance-trained athletes. High-intensity exercise is characterized by the predominance of extramitochondrial pathways of energy production (i.e., phosphagen system and glycolysis), which drive the production and accumulation of hydrogen ions and decrease the pH within the skeletal muscle fibers. These transporters act on the regulation of lactate and hydrogen ions across the cell, and thereby contribute critically to the regulation of intracellular pH and fatigue resistance. In a total of 85 triathletes, the MCT1 genotype was overrepresented in comparison to the genotypic frequency of the general population. However, surprisingly, no significant association was found between this MCT1 genotype and performance mechanical indices such as peak or mean power output measured during a 30-s Wingate sprint in these triathletes [2]. Taken together, these results suggest that at the population-wide level, the prevalence of key genetic polymorphisms may indicate greater fitness or ability to respond to training, but inter-individual variability as well as other epigenetic modifications and psychophysiological and biomechanical factors may confound their association with sport performance among a group of athletes. While the quest to understand the relationship between the inheritance of a trait and performance is relevant and will progress as researchers map out increasingly more of the entire human genome, one must remember that performance is multifaceted.

Performance can be altered drastically by targeted nutritional interventions. In fact, both blood and muscle buffers have been demonstrated to be efficient when it comes to improving high-intensity exercise capacity. However, to date, the impact of nutritional ergogenic aids has mainly been examined during acute and chronic exercise, and scarce data exists during training programs that combine both endurance and strength-type exercises (i.e., concurrent training). A paper published in the current Special Issue by a Brazilian team investigated the effects of a month of beta-alanine (β-alanine) supplementation on the acute interference effect of high-intensity intermittent exercise on lower-body resistance exercise performance, body composition, and strength when combined with a resistance training program [3]. Participants followed a resistance training program based on sets of 10–12 repetitions maximum, four times per week, and were supplemented with either 6.4 g/kg body mass of β-alanine or a placebo. The results demonstrated that β-alanine and placebo supplementation equally increased leg press volume post-training after the high-intensity intermittent exercise, compared to pre-supplementation, and that there were no differences between groups for total training volume or weekly increases in training volume. The authors concluded that chronic β-alanine supplementation did not prevent acute strength loss during resistance exercise after high-intensity intermittent exercise, nor did it increase strength or hypertrophic adaptations associated with resistance training.

The last two papers published in this Special Issue investigated the effect of the acute and chronic application of blood-flow restriction on exercise capacity [4,5]. Restricting blood, and hence oxygen delivery, may sound counterintuitive, but several recent robust studies have demonstrated that, to the contrary, transient restriction that confers an optimal conditioning stimulus could be beneficial to physiological responses and physical endeavours. The restriction can be performed during exercise (the so-called blood-flow restriction modality, BFR) or at rest (this technique has been called ischemic preconditioning, IPC). Fortin and Billaut [4] applied the BFR modality, for the first time, during warm-up to acutely enhance the oxidative response and subsequent sprint endurance performance in well-trained team-sport athletes. The American football players exhibited greater blood volume in the quadriceps and faster muscle reoxygenation (as derived from near-infrared spectroscopy, NIRS) during the recovery periods between 20-m all-out running sprints after applying BFR during their warm-up routine compared to placebo compression. However, while skeletal muscle hemodynamics were positively altered by this innovative warm-up strategy, these physiological changes did not improve short-term repeated-sprint ability. Only a tendency for sprint endurance improvement was noted towards the latter sprints, which made authors speculate that this enhanced warm-up could be beneficial to players during longer activities such as games.

Transient blood-flow occlusion and reperfusion cycles induce severe tissue hypoxia followed by vasodilation, which both upregulate physiological functions acutely through the release of autacoids, changes in ion channel permeability, and changes in the post-translational modifications of proteins, as well as through complex chronic gene transcription and de novo protein synthesis. In fact, IPC has been demonstrated to increase performance during varied types of exercises. The novelty brought forward by the research team from Laval University [5] was to apply IPC before training sessions to enhance the stimulus of every session. Bouffard and colleagues reported that cyclists who preceded their sprint training sessions with IPC for four weeks exhibited a lower decline in power output during a Wingate test than those training without the extra occlusion stimulus. This lower performance decrement was associated with changes in skeletal muscle electrical activity.

## Conclusions

In this ever-changing context of high-performance sports, it is essential to provide practitioners with new evidence-based information about effective stimuli that enhance the efficacy of HIT for varied athletic populations. The original research studies published in this Special Issue have shown that (1) practitioners should employ valid and reliable models and modalities when prescribing HIT sessions, and (2) provided they can handle the extra load, athletes can enhance their chronic responses to exercise with extra physiological stimuli of varied natures.
